# Supplementary material for: Targeting Translation and the Cell Cycle Inversely Affects CTC Metabolism but Not Metastasis
Source: Cancers (Basel). 2023 Nov 2;15(21):5263. doi: 10.3390/cancers15215263 (PMC10650766; doi:10.3390/cancers15215263)
Supplement: Supplementary file 1 [file cancers-15-05263-s001.zip › cancers-2660125-supplementary.pdf]

Figure S1

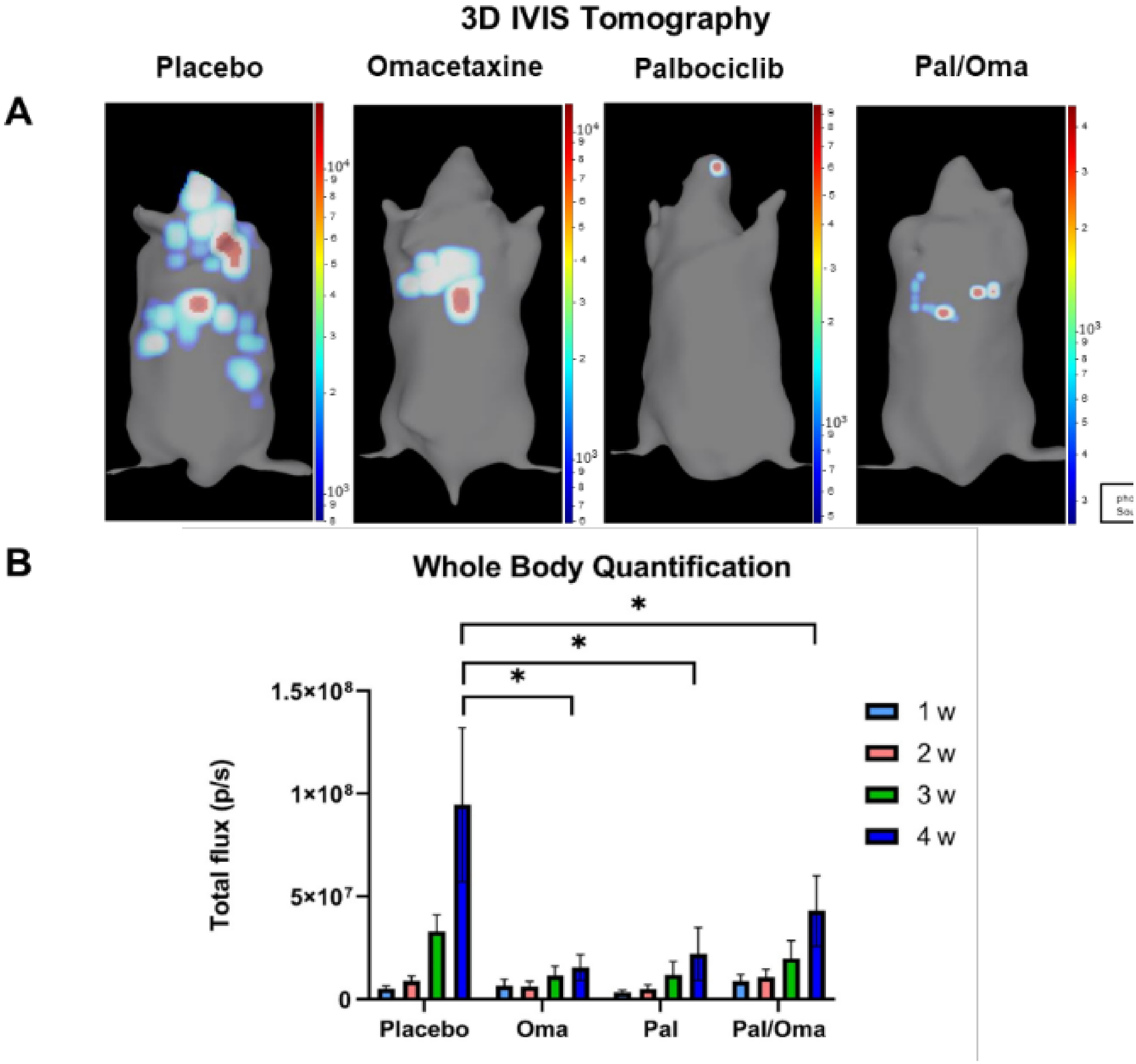
3D IVIS Tomography
Placebo
Omacetaxine
Palbociclib
Pal/Oma
A
B
Whole Body Quantification
*
*
*
1.5×10⁸
1×10⁸
5×10⁷
0
Total flux (p/s)
Placebo
Oma
Pal
Pal/Oma
1 w
2 w
3 w
4 w

Figure S1.

Assessment of mouse metastatic patterns by IVIS imaging. **A**, 3D IVIS tomography of representative mice showed metastases in each animal group. Omacetaxine-treated mice did not have MBM signal. **B**, Total flux of the whole mouse body was measured at different time points (1-4 weeks).

Figure S2

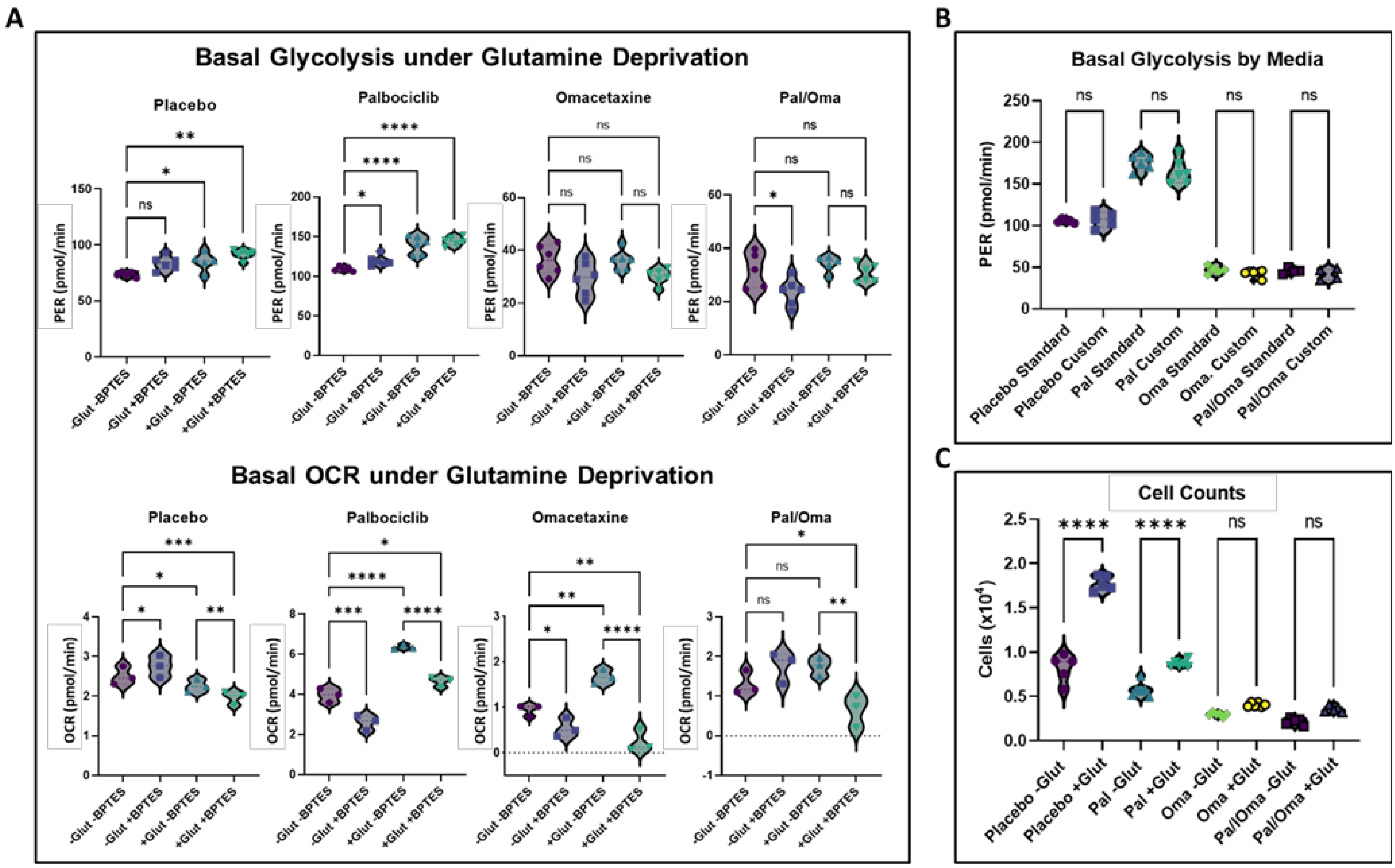
A
Basal Glycolysis under Glutamine Deprivation
Placebo
Palbociclib
Omacetaxine
Pal/Oma
PER (pmol/min)
-Glut -BPTES
-Glut +BPTES
+Glut -BPTES
+Glut +BPTES
Basal OCR under Glutamine Deprivation
OCR (pmol/min)
B
Basal Glycolysis by Media
PER (pmol/min)
Placebo Standard
Placebo Custom
Pal Standard
Pal Custom
Oma Standard
Oma. Custom
Pal/Oma Standard
Pal/Oma Custom
C
Cell Counts
Cells (x10⁴)
Placebo -Glut
Placebo +Glut
Pal -Glut
Pal +Glut
Oma -Glut
Oma +Glut
Pal/Oma -Glut
Pal/Oma +Glut

Figure S2.

Glutamine deprivation experiments in the presence/absence of glutamine and glutaminase inhibitor BPTES (10 μM). For glutamine deprivation experiments, BPTES was added for the entire 3-day treatment course with omacetaxine and/or palbociclib. **A**, Basal Glycolysis and Basal OCR during glutamine deprivation, BPTES, and/or inhibitor treatment. Basal Glycolysis data represent glycoPER output by the Glycolytic Rate Assay and Agilent Wave software, and Basal OCR represents by treatment-group averages of the three pre-first injection OCR reads taken from the Measures sheet of the GRA Report Generator. **B**, comparison of standard cell culture medium with fully supplemented custom media, including glutamine, for confirmation that overall media formulation *per se* could not account for differences seen under glutamine deprivation. **C**, cell counts by inhibitor treatment in the presence or absence of glutamine supplementation. All analyses were performed by two-way ANOVA with Kruskal-Wallis multiple comparisons test. *p = 0.05, **p = .001, ***p = 0.0001, ****p < 0.0001.

Figure S3

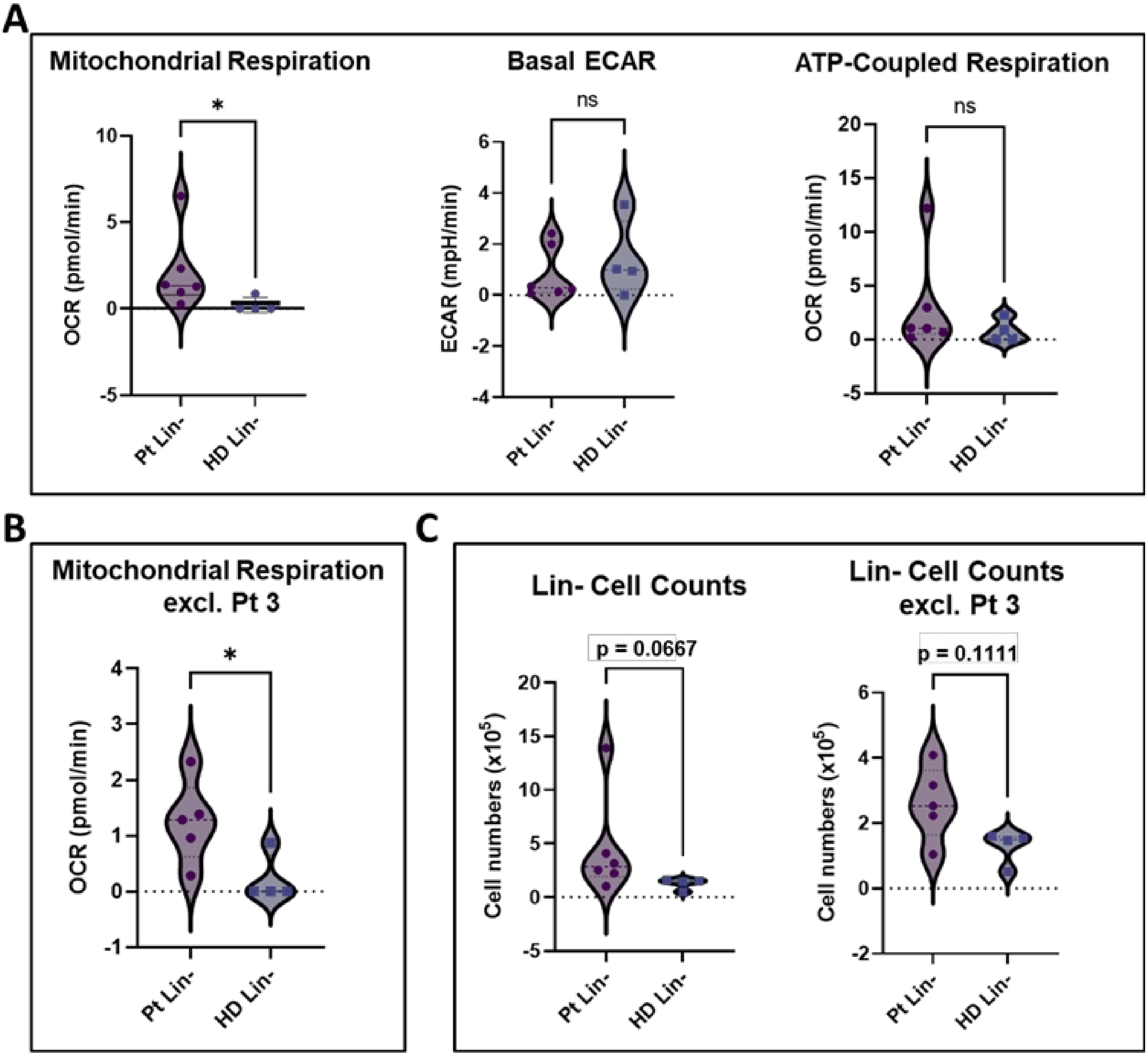

A
Mitochondrial Respiration
*
OCR (pmol/min)
10
5
0
-5
Pt Lin-
HD Lin-
Basal ECAR
ns
ECAR (mpH/min)
6
4
2
0
-2
-4
Pt Lin-
HD Lin-
ATP-Coupled Respiration
ns
OCR (pmol/min)
20
15
10
5
0
-5
Pt Lin-
HD Lin-
B
Mitochondrial Respiration
excl. Pt 3
*
OCR (pmol/min)
4
3
2
1
0
-1
Pt Lin-
HD Lin-
C
Lin- Cell Counts
p = 0.0667
Cell numbers (x10⁵)
20
15
10
5
0
-5
Pt Lin-
HD Lin-
Lin- Cell Counts
excl. Pt 3
p = 0.1111
Cell numbers (x10⁵)
6
4
2
0
-2
Pt Lin-
HD Lin-

Figure S3.

A, Mitochondrial Respiration, Basal ECAR, and ATP-coupled Respiration of patient blood Lin-/CTC fractions versus those from healthy donors, showing significant elevation of mitochondrial respiration in patient Lin-/CTC fractions. **B**, Mitochondrial Respiration of patient Lin-/CTCs versus healthy donor Lin-/CTC fractions excluding patient 3's large and metabolically active Lin-/CTC fraction, showing that significance of the effect is robust even without this patient. **C**, cell counts from all analyzed Lin-/CTC fractions, showing a non-significant trend in the full dataset. All data represent analyses by 2-way unpaired Mann Whitney Test. *p = 0.05, **p = .001, ***p = 0.0001.

Figure S4

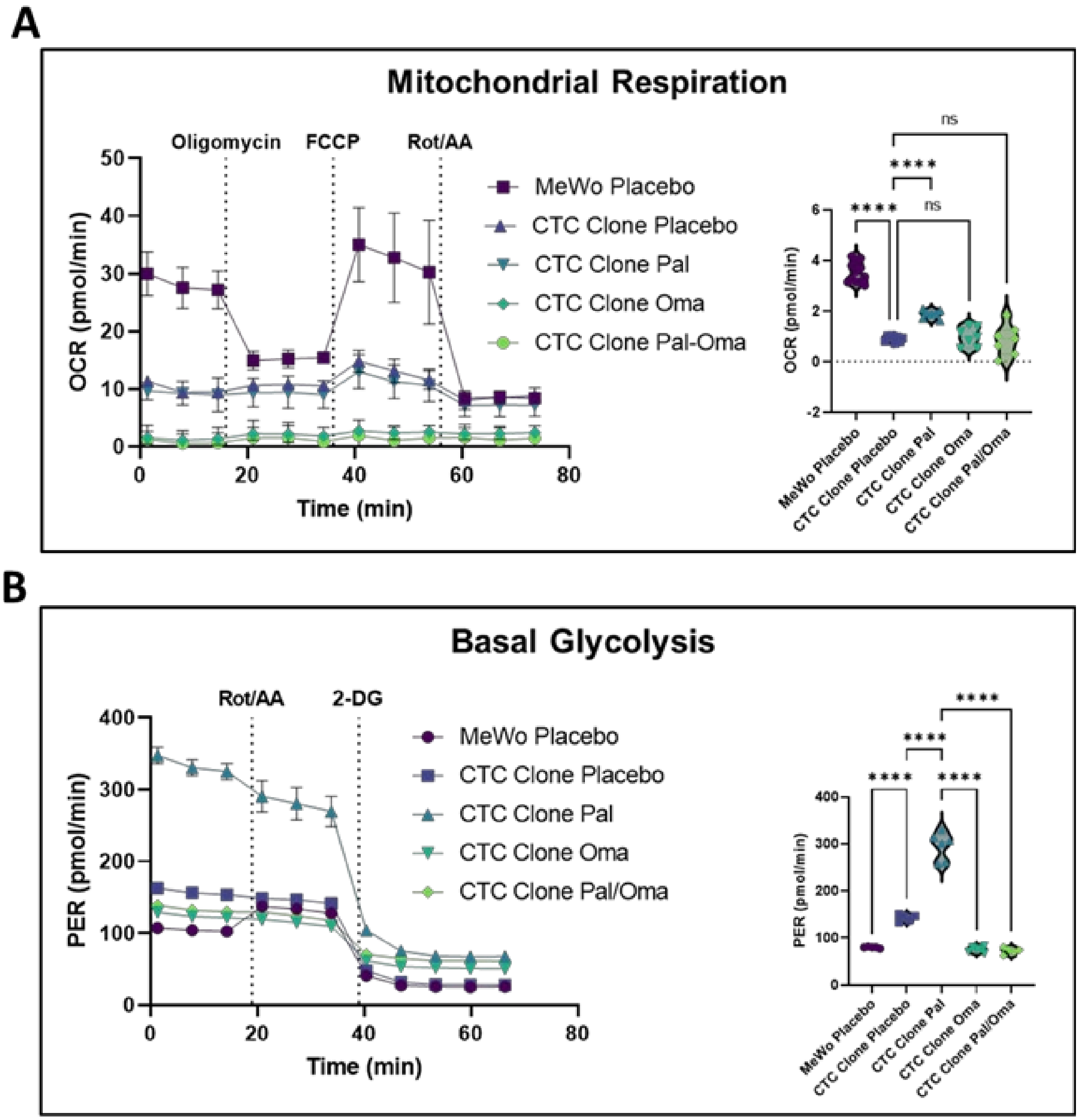
A
Mitochondrial Respiration
Oligomycin
FCCP
Rot/AA
OCR (pmol/min)
Time (min)
MeWo Placebo
CTC Clone Placebo
CTC Clone Pal
CTC Clone Oma
CTC Clone Pal-Oma
ns
****
****
ns
MeWo Placebo
CTC Clone Placebo
CTC Clone Pal
CTC Clone Oma
CTC Clone Pal/Oma
B
Basal Glycolysis
Rot/AA
2-DG
PER (pmol/min)
Time (min)
MeWo Placebo
CTC Clone Placebo
CTC Clone Pal
CTC Clone Oma
CTC Clone Pal/Oma
****
****
****
****
MeWo Placebo
CTC Clone Placebo
CTC Clone Pal
CTC Clone Oma
CTC Clone Pal/Oma

Figure S4.

Baseline metabolic flux results in the melanoma CTC-derived clone. **A**, Mito Stress Test kinetics and Mitochondrial Respiration, and **B**, Glycolytic Rate Assay kinetics and Basal Glycolysis comparing MeWo controls with inhibitor-treated CTC clone. Right panels represent two-way ANOVA with Kruskal-Wallis multiple comparisons test. *$p = 0.05$, **$p = .001$, ***$p = 0.0001$, ****$p < 0.0001$.

Table S1

Statistical analyses for IVIS flux quantification of melanoma brain metastasis

	1 w Mean	1 w SD	1 w N	2 w Mean	2 w SD	2 w N	3 w Mean	3 w SD	3 w N	4 w Mean	4 w SD	4 w N
Placebo	373000	169682.6	5	687000	332334.8	5	2150000	1092196	5	9360000	2975797	5
Oma	139000	9759.61	5	132000	14387.53	5	105000	16672.33	5	115000	6045.475	5
Pal	331000	252049.8	5	504000	387676	5	855000	725114.4	5	2000000	1216133	5
Pal/Oma	130000	20816.36	5	125000	15436.97	5	236000	98212.77	5	321000	45413.74	5

Table S1.

Statistical analyses for IVIS flux quantification of melanoma brain metastasis. The data is presented as a bar graph in Figure 1B. Mean, SD, and number of mice are shown. Mean, SD, and number of mice are shown.

Table S2

Statistical analyses for IVIS flux quantification of extracranial metastasis

	1 w Mean	1 w SD	1 w N	2 w Mean	2 w SD	2 w N	3 w Mean	3 w SD	3 w N	4 w Mean	4 w SD	4 w N
Placebo	5190000	1333679	5	8970000	2309401	5	3.31E+07	7909699	5	9.47E+07	3.75E+07	5
Oma	6550000	3233162	5	6180000	2471059	5	1.14E+07	4838195	5	1.54E+07	6235383	5
Pal	3390000	1212436	5	5080000	2020726	5	1.19E+07	6408588	5	2.21E+07	1.29E+07	5
Pal/Oma	8760000	3302444	5	1.07E+07	3810512	5	1.98E+07	8775724	5	4.30E+07	1.71E+07	5

Supplementary Table 2.

Statistical analyses for IVIS flux quantification of extracranial metastasis. The data is presented as a bar graph in Figure 1C. Mean, SD, and number of mice are shown.

Table S3

Top 12 downregulated genes in MBM

Gene	EXP1	EXP2	EXP3	EXP4
ACOXL	5.30	8.05	4.74	5.62
ANKRD33	5.14	5.18	4.71	6.62
C1orf167-AS1	4.96	24.19	5.36	6.62
CABP1	5.14	4.07	4.60	5.62
CHRNA4	4.16	4.99	4.53	5.62
CLEC4G	4.96	10.61	5.90	6.62
GPRC6A	4.96	25.10	5.19	5.03
GRIA2	5.71	7.56	4.25	6.62
PRAMEF18	4.96	8.29	4.90	6.62
RHCG	6.38	4.23	4.30	6.62
VPREB3	4.96	25.98	4.21	6.03
ZSCAN1	5.30	9.86	4.46	6.62

Table S3.

Top 12 downregulated genes in MBM were determined by the four-pronged experimental approach[28]. None of them represented RPL/RPS CTC signature genes. Four out of 12 genes were negative regulators of translation (highlighted in red).

Table S4

Clinical parameters of melanoma patients

Patient ID	Gender	Age	Stage	Mutation Status	Metastatic Site	Treatment
Metastatic patient 1	Male	73	T2b N2a III-B	ATM Q218, AXL R368Q, AXL R295W, CDKN2A R80, CSF1R W58, GRM3 S154F, GRM3 G18E	Lung	Ipilumumab, Nivolumab
Metastatic patient 2	Male	50	IV	GNA11 mutation, MYC amplification, and SF381 mutation	Neck Lymph Nodes and Liver	Paclitaxel, Carboplatin, Pembrolizumab
Metastatic patient 3	Male	64	IV	None	Parotid mass	Nivolumab
Seahorse patient 1	Male	46	IV	BRAF V600E	Lung, Brain	Nivolumab
Seahorse patient 2	Female	37	IV	None	Chest Wall	None listed
Seahorse patient 3	Male	42	IV	BRAF V600E	Neck, Lymph Nodes, Pancreas	Dabrafenib, Trametinib, Ipilimumab
Seahorse patient 4	Male	65	IV	None	Lung, Brain	Nivolumab, Ipilimumab
Seahorse patient 5	Male	48	IV	None	Neck, Chest, Abdomen, Pelvis	Nivolumab, Ipilimumab

Table S4.

Clinico-pathological characteristics of melanoma patients used in this study. Clinical characteristics included gender, age, stage of disease, mutation status, metastatic sites, and previous/current treatments.

Table S5

Individual values (cpm) of proliferation markers

	Placebo	Omacetaxine	Palbociclib	Pal/Oma	CTC clone
CDK4	1.00	1.00	2.52	1.00	117.29
MKI67	2.14	3.14	1.75	1.00	1008.20
MYC	**3.05**	**1.71**	**1.77**	**1.00**	**56.56**
MYCL	**2.14**	**1.00**	**1.00**	**2.31**	**1.84**
MYCN	**2.14**	**1.71**	**1.00**	**1.00**	**1.14**
PCNA	**1.91**	**1.00**	**1.00**	**1.00**	**264.06**
E2F1	2.14	2.42	3.29	1.65	58.73
E2F2	6.01	6.70	20.04	4.27	31.35
E2F3	**86.20**	**16.70**	**21.42**	**51.31**	**67.06**
E2F4	**5.10**	**1.00**	**1.75**	**3.86**	**69.80**
E2F5	1.91	2.43	2.50	1.00	11.14
E2F6	1.00	3.14	1.00	2.31	10.25
E2F7	**8.25**	**41.59**	**1.77**	**3.12**	**60.73**
E2F8	**2.14**	**1.00**	**1.00**	**3.04**	**18.52**
TK1	1.00	1.00	1.77	1.65	188.52

Table S5.

Individual and mean values (in cpm) of proliferation biomarkers were analyzed. *MYC, MYCL, MYCN, PCNA, E2F3, E2F4, E2F7, E2F8* gene expression were downregulated in response to treatment with palbociclib (n=5).
